# Bidirectional Association between Hypertension and NAFLD: A Systematic Review and Meta-Analysis of Observational Studies

**DOI:** 10.1155/2022/8463640

**Published:** 2022-03-24

**Authors:** Gerui Li, Yuanyuan Peng, Ze Chen, Hang Li, Danli Liu, Xujun Ye

**Affiliations:** ^1^Department of Geriatrics, Zhongnan Hospital of Wuhan University, Wuhan 430071, Hubei, China; ^2^Department of Cardiology, Zhongnan Hospital of Wuhan University, Wuhan 430071, Hubei, China

## Abstract

An increasing body of evidence connects non-alcoholic fatty liver disease (NAFLD) to hypertension. The objective of this systematic review and meta-analysis was to estimate the nature and magnitude of the association between NAFLD and hypertension. We systematically searched PubMed, Embase, Cochrane Library, and Web of Science for observational studies published up to May 1, 2021. Cohort studies that reported data on the association between NAFLD and incident hypertension or between hypertension and incident NAFLD were included. We used random-effects models to conduct meta-analysis on the measures of association from individual studies. A total of 11 studies were eligible for inclusion, among which 4 studies including 25,260 participants reported the association between hypertension and new-onset NAFLD. The presence of hypertension was significantly associated with an increased risk of incident NAFLD (HR 1.63, 95% CI: 1.41–1.88; *I*^2^ = 37.6%). On the other hand, 9 studies with data on 46,487 participants analyzed the effects of NAFLD on incident hypertension. Pooled analysis showed that the presence of NAFLD was significantly associated with an increased incidence of hypertension (HR 1.55, 95% CI: 1.29–1.87; *I*^2^ = 80.5%). There was significant heterogeneity among the studies in this analysis (*p* < 0.01). Sensitivity analyses showed that the magnitude of the association was significantly different in subgroups stratified by a mean age of participants and geographical location, which explains part of the heterogeneity. In conclusion, this meta-analysis indicates the existence of a bidirectional relationship between NAFLD and hypertension independent of traditional cardiometabolic risk factors.

## 1. Introduction

Nonalcoholic fatty liver disease (NAFLD) encompasses a pathological spectrum ranging from simple steatosis through steatohepatitis (NASH) to advanced fibrosis, cirrhosis, and ultimately hepatocellular carcinoma [1, 2]. NAFLD has emerged as the most common liver and metabolic disease plaguing the world, with a global prevalence of about 25% in 2018 (i.e., 1.7 billion individuals). The number of individuals with NAFLD even surpasses the combined population with diabetes mellitus (400 million) and obesity (650 million) [1, 3]. NAFLD has traditionally been considered as the simple “hepatic manifestation” of metabolic syndrome. Obesity, type 2 diabetes, hyperlipidemia, and insulin resistance have been established as risk factors for NAFLD [4]. However, the association between NAFLD and metabolic syndrome components is much more complex and even closer than previously thought [5]. Indeed, as an underestimated metabolic abnormality, NAFLD is now recognized as a multisystem disease that is strongly associated with an increased risk of a wide range of extrahepatic complications such as cardiovascular disease (CVD) [1, 4].

Hypertension (HTN), a major component of metabolic syndrome affecting around 30% of the general population, has long been recognized as one of the strongest risk factors for CVD [6, 7]. Emerging evidence suggests that elevated blood pressure levels, even within the normal range, could predict the onset of NAFLD [8–11]. On the other hand, the presence and severity of NAFLD are also reported to be correlated with the incidence of HTN [8, 9, 12–19]. Collectively, these new lines of evidence imply the existence of a possible bidirectional association between HTN and NAFLD [5–8]. However, the power of every single study may not be adequate to draw a solid conclusion. In addition, there are also studies that showed a non-significant association after multiple adjustments [8, 12, 13]. Thus, the possible bidirectional relationship between NAFLD and HTN and the real effect size and power of this association remain largely unknown.

Given the huge number of patients with NALFD and HTN worldwide, elucidating their relationship is important for risk prediction, primary prevention, and exploring therapeutic regimens for these two diseases. We therefore attempted a synthesis of the available evidence from observational cohort studies under a meta-analysis to examine the relationship between NAFLD and HTN and to assess the magnitude of this association.

## 2. Methods

### 2.1. Data Sources and Searches

We did a systematic literature search of PubMed, Embase, Cochrane Library, and Web of Science for the identification of articles reporting the results of longitudinal observational studies published up to May 1, 2021, investigating the associations of NAFLD on incident HTN and/or of HTN on incident NAFLD. We used a combined text and medical subject headings (MeSH) search strategy with the terms: “Nonalcoholic Fatty Liver Disease,” “NAFLD,” “Non alcoholic Fatty Liver Disease,” “Nonalcoholic Fatty Liver,” “Nonalcoholic Fatty Livers,” “Liver, Nonalcoholic Fatty,” “Livers, Nonalcoholic Fatty,” “Fatty Liver, Nonalcoholic,” “Fatty Livers, Nonalcoholic,” “Nonalcoholic Steatohepatitis,” “Nonalcoholic Steatohepatitides,” “Steatohepatitis, Nonalcoholic,” “Steatohepatitides, Nonalcoholic,” “Hypertension,” “High Blood Pressure,” “High Blood Pressures,” “Blood Pressure, High,” and “Blood Pressures, High.” The search was restricted to studies in human beings, and no language restrictions were applied. Full electronic search strategies for PubMed and Embase are shown in Table S1. We also checked the reference lists of potentially relevant original articles and reviews for identifying further eligible studies. This systematic review was conducted by following the Preferred Reporting Items for Systematic Reviews and Meta-Analyses (PRISMA) guidelines [20]. We followed the reporting items proposed by the Meta-Analysis of Observational Studies in Epidemiology (MOOSE) for the meta-analysis due to the observational nature of the included studies [21].

### 2.2. Study Selection

Studies that met the following criteria were included: (1) longitudinal design; (2) participants aged ≥18 years; (3) follow-up duration ≥2 years; (4) assessment of the association between NAFLD and incident HTN and/or between HTN and incident NAFLD; (5) reported data on hazard ratios (HRs) or odds ratios (ORs) with confidence intervals (CIs) for the outcome of interest, and the estimate was at least adjusted for age and gender; and (6) NAFLD diagnosed by aminotransferase levels, surrogate scores, imaging methods, or liver biopsy and HTN diagnosed based on blood pressure. Criteria for exclusion of selected articles were as follows: (1) cross-sectional studies, (2) studies that were conducted in the pediatric population, (3) studies with a median follow-up less than 2 years, (4) studies did not report the adjusted point estimate, and (5) review articles, case reports, practice guidelines, commentaries or editorials. Two authors (GL and YP) independently assessed all titles and abstracts and reviewed full texts of potentially relevant articles for inclusion. Any disagreement was resolved by consensus, referring back to the original paper.

### 2.3. Data Extraction and Quality Evaluation

All data were extracted into a prepiloted data extraction form developed in Excel. For all included studies, we extracted information on study design, country/region, follow-up duration, diagnostic methods of NAFLD and HTN, the outcome of interest, risk estimates (95% CI), and adjustment factors. The most adjusted estimate in the multivariable regression model was extracted when a study presented more than one risk estimate. In the case of multiple articles on the same subjects, we included only the latest and most comprehensive one. Two authors (GL and YP) conducted quality evaluation independently, and any disagreement in rating was addressed by discussion. The Newcastle–Ottawa Scale (NOS) was applied for the quality evaluation of included cohort studies. The quality of each study ranges from 1 to 9 points based on three domains: the cohort selection (maximum of 4 points), the comparability of the cohort design and analysis (maximum of 2 points), and the adequacy of the outcome measures (maximum of 3 points). Studies achieving a score of at least seven points were considered high quality [22].

### 2.4. Data Synthesis and Statistical Analysis

For assessing the effect of NAFLD on the risk for incident HTN, the effect size was estimated as HRs or ORs with 95% CIs, according to NAFLD patients versus non-NAFLD patients shown in each included study, using non-NAFLD patients as the reference group. For evaluating the effect of HTN on the risk for incident NAFLD, the effect size was estimated as HRs or ORs with 95% CIs, according to HTN patients versus non-HTN patients reported in each eligible study, using non-HTN patients as the reference. The results were pooled, and an overall estimate of effect size was calculated using a random-effects model to take into account the heterogeneity among studies, as we expected a relatively large heterogeneity in our pooled results of observational studies on different cohorts with varying degrees of adjustment. The Q-statistic and the *I*-squared (*I*^2^) statistic were applied to assess the heterogeneity across studies. Large *I*^2^ (>50%) or *p* < 0.1 for Q-statistic suggests substantial heterogeneity across studies.

A funnel plot showing the logarithm of the effect measure against the logarithm of its standard error was constructed to evaluate the presence of publication bias. We also applied both the Egger's test [23] and the rank correlation Begg's test [24]. To evaluate the possible sources of heterogeneity and the robustness of our findings, we performed prespecified subgroup-sensitivity analyses by the methodology used to diagnose NAFLD, mean age of participants, geographical location, and follow-up duration. Moreover, additional sensitivity analyses were performed by removing each individual study from the meta-analysis at a time, to investigate whether the pooled effect estimate was substantially influenced by a single specific study. All statistical analyses were done with Stata Version 15.0 software (Stata Corp, College Station, TX). A two-tailed *p*-value <0.05 was considered significant.

## 3. Results

### 3.1. Characteristics of Included Studies and Participants

**Figure 1:**
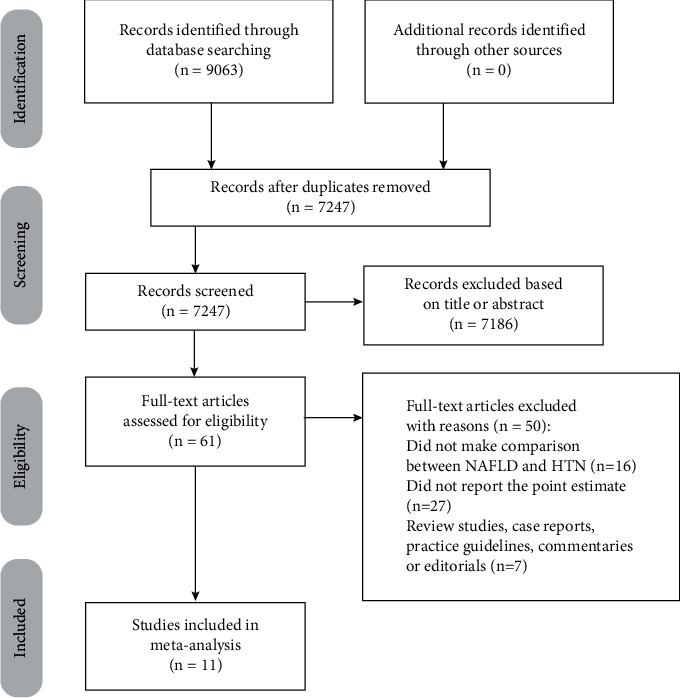
Flow chart of article selection.

As specified in the PRISMA flow diagram (Figure 1), our initial search yielded 9,063 records, of which 7,247 remained after removing duplicates. We initially identified 61 potentially eligible studies in accordance with the aforementioned inclusion and exclusion criteria and further excluded 50 articles after reviewing the full texts. Finally, a total of 11 unique studies were eligible for inclusion in our meta-analysis and were evaluated for quality [8–18], among which 2 studies assessed the association between NAFLD and incident HTN and the association between HTN and incident NAFLD simultaneously [8, 9].

The main characteristics of the 11 included studies are summarized in Table 1. Eight studies were conducted in Asia (3 in China, 1 in Japan, and 4 in South Korea), 2 in Europe (1 in France and 1 in Germany), and 1 in the United States. One study [12] was performed only in males, while all the rest included both male and female individuals. NAFLD was diagnosed by ultrasound in 6 studies, by CT images in 1 study, and by surrogate scores (fatty liver index (FLI), hepatic steatosis index, and comprehensive NAFLD score) in 4 studies. The mean follow-up duration of included studies ranges from 2.6 years to 11.6 years. All studies adjusted age and sex, and most studies further adjusted traditional cardiometabolic risk factors such as blood pressure, diabetes, dyslipidemia, and obesity. Nine studies with data on 46,487 participants assessed the association between preexisting NAFLD and new-onset HTN, whereas 4 studies with data on 25,260 participants investigated the association between established HTN and incident NAFLD. All included cohort studies were considered as high quality (Table 1).

### 3.2. Association between HTN and Incident NAFLD

**Figure 2:**
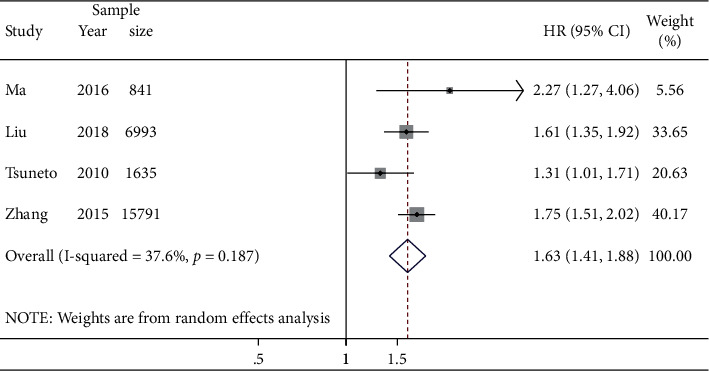
HTN versus non-HTN on the risk of incident NAFLD. Forest plot and pooled estimates of 4 eligible studies with data on 25,260 participants.

Pooling the adjusted effect estimates from individual studies indicated that the presence of HTN was significantly associated with an increased risk of incident NAFLD (HR 1.63; 95% CI: 1.41–1.88; *n* = 4 studies, 25,260 participants). The test for heterogeneity among the individual studies was not significant (*I*^2^ = 37.6%, *p* = 0.187; Figure 2). Asymmetry analysis in the funnel plot showed no evidence of significant publication bias, and both Egger's test (*p* = 0.987) and rank correlation Begg's test (*p* = 0.734) did not show statistically significant asymmetry (Figure S1). In the leave-one-out analyses, the significantly increased risk was not materially changed, with pooled HRs ranging from 1.56 (95% CI: 1.25–1.93) to 1.71 (95% CI: 1.53–1.91; Figure S2).

### 3.3. Association between NAFLD and Incident HTN

**Figure 3:**
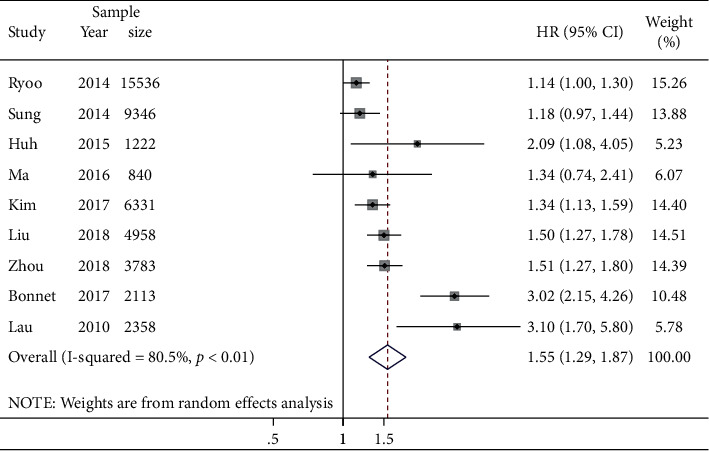
NAFLD versus non-NAFLD on the risk of incident HTN. Forest plot and pooled estimates of 9 eligible studies with data on 46,487 participants.

**Figure 4:**
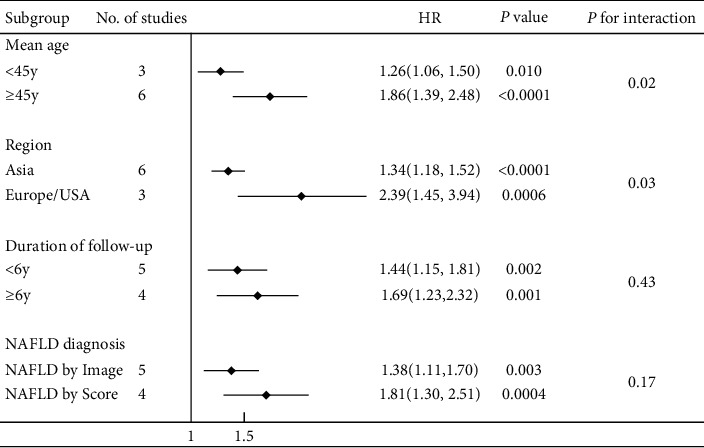
Subgroup sensitivity analyses on studies assessing the association between NAFLD and incident HTN, stratified based on the mean age of participants, geographical regions, duration of follow-up, and NAFLD diagnostic methods, respectively.

Pooled results indicated that the presence of NAFLD was significantly associated with an increased risk of incident HTN (pooled HR 1.55; 95% CI: 1.29–1.87; *n* = 9 studies, 46,487 participants; Figure 3). Potential heterogeneity was observed among the individual studies (*I*^2^ = 80.5%, *p* < 0.01). We conducted subgroup analyses to explore potential sources of heterogeneity (Figure 4). Subgroup analysis stratified by NAFLD diagnostic methods showed that compared with the studies where NAFLD was diagnosed by imaging methods (HR 1.38, 95% CI: 1.11–1.70; *n* = 5 studies), a higher risk was seen in studies where NAFLD was diagnosed by surrogate scores (HR 1.81, 95% CI: 1.30–2.51; *n* = 4 studies). However, no significant results were found in the test for differences between subgroups stratified by NAFLD diagnostic methods (*p* = 0.17) as well as the follow-up duration (*p* = 0.43). In contrast, a significant between-group heterogeneity was found in subgroup analyses stratified by mean age of participants (*p* = 0.02) and geographical region (*p* = 0.03). Asymmetry analysis in the funnel plot showed no evidence of significant publication bias, and both Egger's test (*p* = 0.062) and rank correlation Begg's test (*p* = 0.118) did not show statistically significant asymmetry (Figure S3). We also conducted a sensitivity analysis to test for the possibility of the excessive influence of individual studies by eliminating each of the included studies one at a time. The significantly increased risk was not substantially changed in the leave-one-out analyses, with pooled HRs ranging from 1.39 (95% CI: 1.21–1.61) to 1.64 (95% CI: 1.35–2.00; Figure S4).

## 4. Discussion

In this meta-analysis of 11 observational cohort studies, we found that the presence of NAFLD is significantly associated with a higher risk of incident HTN (HR 1.55, 95% CI: 1.29–1.87; *I*^2^ = 80.5%; *n* = 9 studies, 46,487 participants). On the other hand, the presence of HTN was significantly associated with a higher incidence of NAFLD (HR 1.63, 95% CI: 1.41–1.88; *I*^2^ = 37.6%; *n* = 4 studies, 25,260 participants). The results from longitudinal cohort studies clearly suggest a bidirectional relationship between NAFLD and HTN, and this association appears to be independent of traditional cardiometabolic risk factors. The close two-way association between the two entities can conceivably form a vicious circle during disease progression. It is therefore important to increase public awareness of the notion that NAFLD is an independent risk factor and might serve as a driving force in the development and progression of HTN and vice versa.

The findings of this meta-analysis confirm and extend the results of a previous smaller meta-analysis published in 2016 [4]. In that meta-analysis, Wu et al. incorporated only three studies and reported that NAFLD was associated with a nearly 16% increased risk of incident hypertension (HR 1.16, 95% CI: 1.06–1.27), with potential heterogeneity among the studies (*I*^2^ = 55.9%, *p* = 0.059). Compared with the meta-analysis by Wu et al., we have increased the number of eligible studies to 9 in the analysis of the association between NAFLD and incident hypertension. Moreover, we further analyzed the association between hypertension and incident NAFLD, thus enabling the investigation of a possible bidirectional relationship.

In light of our results and given the extremely high prevalence of both NAFLD and HTN in the general population, identifying NAFLD as an emerging risk factor for HTN and vice versa may help improve the risk prediction, identify primary preventive strategies, and select therapeutic regimens for the two diseases and their related complications [7, 19]. Indeed, two studies have investigated the effects of alteration in NAFLD status over time on the risk of developing HTN [9, 13]. While incident NAFLD was significantly associated with new-onset HTN (HRs 1.49 (95% CI: 1.26, 1.76) and 1.36 (95% CI: 1.10, 1.67), respectively), no significant association was found between resolutive NAFLD and incident HTN (HRs 1.16 (95% CI: 0.96, 1.39) and 1.21 (95% CI: 0.90, 1.63), respectively), suggesting that NAFLD management may serve as a potentially important aspect in the prevention of HTN. On the other hand, one study reported the HR for incident NAFLD in people with controlled HTN was 0.74 (95% CI: 0.53–1.03), suggesting that blood pressure control may help reduce the risk of developing NAFLD [9]. It is also suggested that patients with proven NAFLD should receive effective blood pressure control, which may help attenuate the progression to liver fibrosis [6].

**Figure 5:**
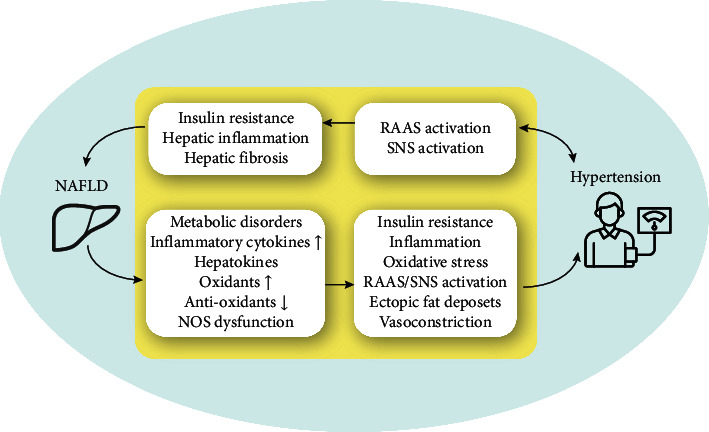
Putative mechanisms through which NAFLD may drive the development of HTN and vice versa.

Despite the growing body of clinical evidence linking NAFLD to HTN, the mechanisms underpinning this association has yet to be identified (Figure 5) [5–7]. In the presence of NAFLD, several alterations occur in the liver that may increase the risk of HTN, such as dysregulation of glucose and lipid metabolism, disturbance of immunologic homeostasis, and increased release of proinflammatory, profibrinogen, and prooxidant molecules (e.g., cytokines, hepatokines, and oxidants). These alterations further promote insulin resistance, inflammation, and oxidative stress both at the systemic and local (e.g., vascular and renal) level; stimulate the activation of the sympathetic nervous system (SNS) and renin-angiotensin-aldosterone system (RAAS); and increase ectopic fat deposits (e.g., perivascular fat and renal sinus fat) [7]. In addition, NAFLD may directly increase vasoconstriction and decrease vasodilation by regulating the function of nitric oxide synthase (NOS) [7]. All these NAFLD-related pathophysiological changes may predispose to the development of HTN [19]. On the other hand, RAAS plays a major role in the pathogenesis of HTN. Recent lines of evidence have indicated a relevant relationship between RAAS and NAFLD, suggesting a shared pathophysiological pathway between the two diseases [6]. Angiotensin II has been shown to promote insulin resistance and contribute to hepatic injury and fibrosis [6]. Moreover, it is reported that SNS activation is also implicated in enhanced liver fibrogenesis in patients with NAFLD [25]. Despite these putative mechanisms, further studies are needed to gain an in-depth understanding of the underlying mechanisms, which may help develop novel and effective therapies.

The present meta-analysis has several limitations. First, the observational nature of the included studies could not allow the interpretation of a causality link between the exposure and the outcome. Second, the study protocol of this systematic review and meta-analysis has not been preregistered. Third, in the present study, the pooled analysis of the association between NAFLD and incident HTN revealed a certain level of heterogeneity. Thus, these results should be interpreted cautiously. The heterogeneity may be derived from a combination of factors including diagnostic methods for NAFLD, age of participants, geographical region, covariate adjustment, and other potential unmeasured confounders. The included studies have defined NAFLD by various diagnostic methods, resulting in variations in their reported outcomes. Our subgroup analysis stratified by NAFLD diagnostic methods showed that compared with the studies where NAFLD was diagnosed by imaging methods (HR 1.38), a higher risk was seen in studies where NAFLD was diagnosed by surrogate scores (HR 1.81), although no significant results were found in the test for differences between the subgroups (*p* = 0.17). Moreover, covariate adjustment and potential unmeasured variables can serve as another important source of heterogeneity. As shown in Table 1, although most of the included studies adjusted the results for several potential confounders including age, sex, cigarette smoke, and other conventional cardiometabolic risk factors, the possibility of residual confounding by some unmeasured factors cannot be excluded. It should be noted that some studies reported incomplete adjustments for well-established risk factors and potential confounders such as baseline BMI, waist circumference, or diabetes. It was therefore not possible to combine models in studies that adjusted for the same set of potential confounders. More detailed analyses of the sources of heterogeneity might require collaborative pooling of participant data at an individual level from large cohort studies. Fourth, none of the eligible studies applied liver biopsy, the gold standard technique, to diagnose NAFLD. Indeed, although liver biopsy is more reliable than both liver ultrasonography and surrogate scores such as FLI, this procedure is expensive, time-consuming, and invasive and carries a high risk of potentially serious complications, such as severe pain, hemorrhage, and transient hypotension, making it unsuitable for large-scale population studies.

Albeit these limitations, our meta-analysis also has several important strengths. To our knowledge, this is the first meta-analysis to directly and specifically analyze the bidirectional association between NAFLD and HTN. In addition, we conducted a comprehensive search, used predetermined inclusion and exclusion criteria, and did a robust quality assessment, which means we have included the best available evidence to report on the association between the two diseases. Moreover, the large number of participants and events involved in our meta-analysis yields adequate statistical power to quantify the association between NAFLD and HTN. Finally, the overall quality of the included studies in the meta-analysis was considered as high, and there was no sign of significant publication bias affecting the results when evaluated by both Egger and Begg's tests.

In conclusion, our meta-analysis clearly suggests the existence of a bidirectional relationship between NAFLD and HTN independent of traditional cardiometabolic risk factors. The presence of NAFLD is significantly associated with a 1.55-fold increased risk of incident HTN, whereas HTN is significantly associated with a 1.63-fold increased risk of incident NAFLD. Recognizing NAFLD as a risk factor for HTN and vice versa is important to improve the risk prediction, identify primary preventive strategies, and explore therapeutic regimens for these two increasingly prevalent and burdensome diseases. More studies in the future should investigate whether changes in NAFLD status over time can modify the risk of developing or worsening HTN and vice versa, thus providing more solid evidence to definitively establish a cause-effect relationship. In addition, further researches are needed to gain an in-depth understanding of the underlying mechanisms and to identify novel and effective treatments.

## Figures and Tables

**Table 1:** Characteristics of 11 observational studies included in the meta-analysis.

Author and year	Country	Study characteristics	Mean follow-up duration	Fatty liver diagnosis	Hypertension diagnosis	Outcomes	Adjustments	Quality score^*∗*^
Ryoo et al., 2014 [12]	South Korea	Population-based; *n* = 22,090 men; mean age 42.1 y; 6,554 mild NAFLD; 1,007 moderate to severe NAFLD; without baseline HTN; 3,820 incident HTN	Prospective cohort; 5 years	Liver ultrasound	SBP ≥ 140 mmHg and/or DBP ≥ 90 mmHg or current use of antihypertensive agents	Association with incident HTN	Age, BMI, TG, serum creatinine, AST, ALT, GGT, recent smoking status, regular exercise, diabetes mellitus	7
Sung et al., 2014 [13]	South Korea	Population-based; *n* = 11,448; mean age 41 y; 7,940 men; 2,958 baseline fatty liver; without baseline HTN; 911 incident HTN	Retrospective cohort; 5 years	Liver ultrasound	SBP ≥ 140 mmHg and/or DBP ≥ 90 mmHg or current use of antihypertensive agents	Association with incident HTN	Age, sex, alcohol consumption, smoking status, exercise, SBP, BMI, diabetes status, GGT, HOMA-IR, eGFR, change in BMI between baseline and follow-up	8
Huh et al., 2015 [14]	South Korea	Population-based; *n* = 1,521; age 40–70 y; 484 men; 124 baseline NAFLD; without baseline HTN; 153 incident HTN	Prospective cohort; 2.6 years	FLI ≥ 60	SBP ≥ 140 mmHg and/or DBP ≥ 90 mmHg or current use of antihypertensive agents	Association with incident HTN	Age, gender, baseline SBP, baseline DBP, smoking, regular exercise, alcohol intake, diabetes, log ALT, log HOMA-IR, hsCRP, serum creatinine, adiponectin	7
Ma et al., 2016 [8]	USA	Population-based; *n* = 1,051; mean age 45 y; 572 men; 187 baseline fatty liver; 82 baseline HTN	Prospective cohort; 6 years	CT	SBP ≥ 140 mmHg and/or DBP ≥ 90 mmHg or current use of antihypertensive agents	Association with incident HTN	Age, sex, baseline current smoking, physical activity, alcohol intake, SBP, DBP, VAT, delta VAT, delta LPR	7
						Association with incident fatty liver		
Kim et al., 2017 [15]	South Korea	Population-based; *n* = 6,331; mean age 51y; 2,995 men; 1,517 baseline NAFLD; without baseline HTN; 891 incident HTN	Retrospective study; 8.7 years	FLI, hepatic steatosis index, and comprehensive NAFLD score; two or three indexes were satisfied	Blood pressure levels	Association with incident HTN	Age, gender, diabetes mellitus, family history of HTN and obesity	7
Liu et al., 2018 [9]	China	Population-based; 6,704 HTN-free subjects; 2,008 baseline NAFLD; 2,561 incident HTN; 9,328 NAFLD-free subjects; 4,436 baseline HTN; 2,289 incident NAFLD	Prospective cohort; 5 years	Liver ultrasound	SBP ≥ 140 mmHg and/or DBP ≥ 90 mmHg or current use of antihypertensive agents or prior diagnosis	Association with incident HTN	Age, sex, past history of CHD, family history of HTN, diabetes, BMI	9
						Association with incident NAFLD		
Zhou and Cen, 2018 [16]	China	Population-based; *n* = 4,687; mean age 40 y; 3,177 men; 304 baseline NAFLD (FLI ≥ 60); without baseline HTN; 2,047 incident HTN	Prospective cohort; 9 years	FLI	SBP ≥ 140 mmHg and/or DBP ≥ 90 mmHg or current use of antihypertensive agents	Association with incident HTN	Age, gender, indicators of metabolic syndrome (waist circumference, SBP, DBP, FPG, HDL-C, TG)	8
Bonnet et al., 2017 [17]	France	Population-based; *n* = 2,565 (insulin resistance cohort); aged 30–65y; 1,138 men; 220 baseline NAFLD (FLI ≥60); without baseline HTN; 1,021 incident HTN	Prospective cohort; 9 years	FLI	SBP ≥ 140 mmHg and/or DBP ≥ 90 mmHg or current use of antihypertensive agents	Association with incident HTN	Age, sex, smoking, FPG, alcohol intake	7
Lau et al., 2010 [18]	Germany	Population-based; *n* = 3,191; 1,532 men; aged 20–79 years	Prospective cohort; 5.3 years	Liver ultrasound and AST	SBP ≥ 140 mmHg and/or DBP ≥ 90 mmHg or current use of antihypertensive agents	Association with incident HTN	Age, sex, waist circumference, BMI, diabetes mellitus, alcohol consumption, use of antihypertensive medication	8
Tsuneto et al., 2010 [10]	Japan	1,635 Nagasaki atomic bomb survivors; 606 men; without baseline fatty liver; 323 incident fatty liver	Retrospective cohort; 11.6 years	Liver ultrasound	SBP ≥ 130 mmHg and/or DBP ≥ 85 mmHg	Association with incident fatty liver	Age, sex, smoking and drinking habits, obesity, hypercholesterolemia, low HDL-C, hypertriglyceridemia, glucose intolerance, atomic radiation dose	7
Zhang et al., 2015 [11]	China	Population-based; *n* = 15,791; mean age 42.5 y; 7,922 men; 877 baseline HTN; without baseline NAFLD; 3,913 incident NAFLD	Prospective cohort; 3.3 years	Liver ultrasound	SBP ≥ 140 mmHg and/or DBP values ≥ 90 mmHg or prior diagnosis	Association with incident NAFLD	Age, gender, smoking status, diet, regular exercise	7

Abbreviations: HTN, hypertension; SBP, systolic blood pressure; DBP, diastolic blood pressure; NAFLD, non-alcoholic fatty liver disease; BMI, body mass index; FLI, fatty liver index; AST, aspartate aminotransferase; ALT, alanine aminotransferase; GGT, glutamyl transpeptidase; HDL-C, high-density lipoprotein cholesterol; hsCRP, high-sensitivity C-reactive protein; TG, triglyceride; FPG, fasting plasma glucose; HOMA-IR, homeostasis model assessment of insulin resistance; VAT, visceral adipose tissue; LPR, liver-phantom ratio; and CHD, coronary heart disease. ^*∗*^The Newcastle–Ottawa Scale (NOS) was applied for the quality evaluation of cohort studies. The quality of each cohort study ranges from 1 to 9 points based on three domains: the cohort selection (maximum 4 points), the comparability of the cohort design and analysis (maximum 2 points), and the adequacy of the outcome measures (maximum 3 points). Studies achieving a score of at least seven points were considered high quality.

## Data Availability

This is a systematic review and meta-analysis.

## Supplementary Materials

Supplementary Materials Figure S1: funnel plot of selected studies (hypertension to incident NAFLD) describing the relationship between effect size and standard error on the log scale. The vertical line represents the pooled effect size and the dashed lines represent the pseudo 95% confidence intervals. Figure S2: sensitivity analysis of the comparison between HTN and non-HTN on the risk of incident NAFLD. Figure S3: funnel plot of selected studies (NAFLD to incident hypertension) describing the relationship between effect size and standard error on the log scale. The vertical line represents the pooled effect size and the dashed lines represent the pseudo 95% confidence intervals. Figure S4: sensitivity analysis of the comparison between NAFLD and non-NAFLD on the risk of incident HTN. Table S1: Search strategy for PubMed and Embase.
