# Supplementary material for: Bidirectional Association between Hypertension and NAFLD: A Systematic Review and Meta-Analysis of Observational Studies
Source: Int J Endocrinol. 2022 Mar 24;2022:8463640. doi: 10.1155/2022/8463640 (PMC8970889; doi:10.1155/2022/8463640)
Supplement: Supplementary Materials — Figure S1: funnel plot of selected studies (hypertension to incident NAFLD) describing the relationship between effect size and standard error on the log scale. The vertical line represents the pooled effect size and the dashed lines represent the pseudo 95% confidence intervals. Figure S2: sensitivity analysis of the comparison between HTN and non-HTN on the risk of incident NAFLD. Figure S3: funnel plot of selected studies (NAFLD to incident hypertension) describing the relationship between effect size and standard error on the log scale. The vertical line represents the pooled effect size and the dashed lines represent the pseudo 95% confidence intervals. Figure S4: sensitivity analysis of the comparison between NAFLD and non-NAFLD on the risk of incident HTN. Table S1: Search strategy for PubMed and Embase. [file 8463640.f1.pdf]

Supplementary data

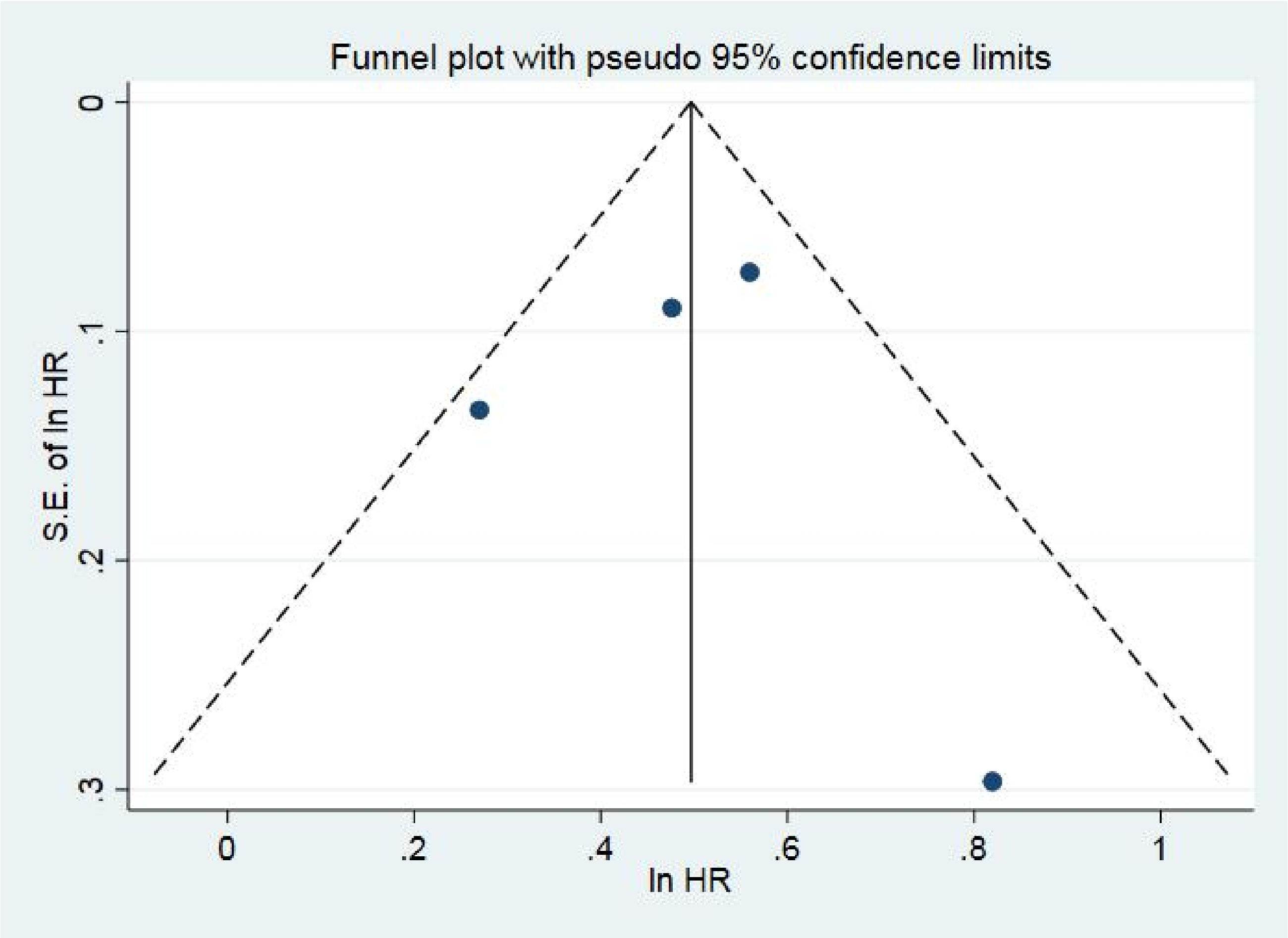

Fig. S1. Funnel plot of selected studies (hypertension to incident NAFLD) describing the relationship between effect size and standard error on the log scale. The vertical line represents the pooled effect size and the dashed lines represent the pseudo 95% confidence intervals.

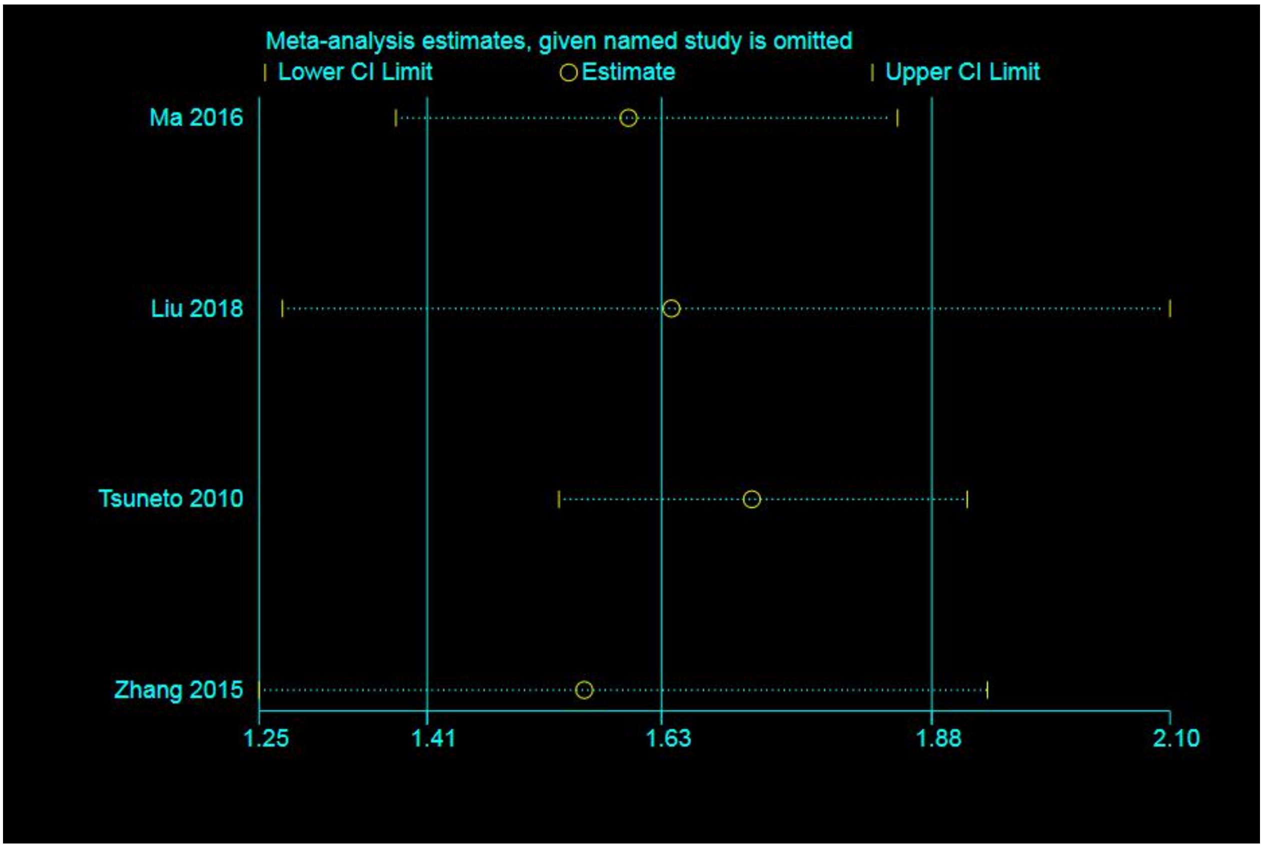

Fig. S2. Sensitivity analysis of the comparison between HTN and non-HTN on the risk of incident NAFLD.

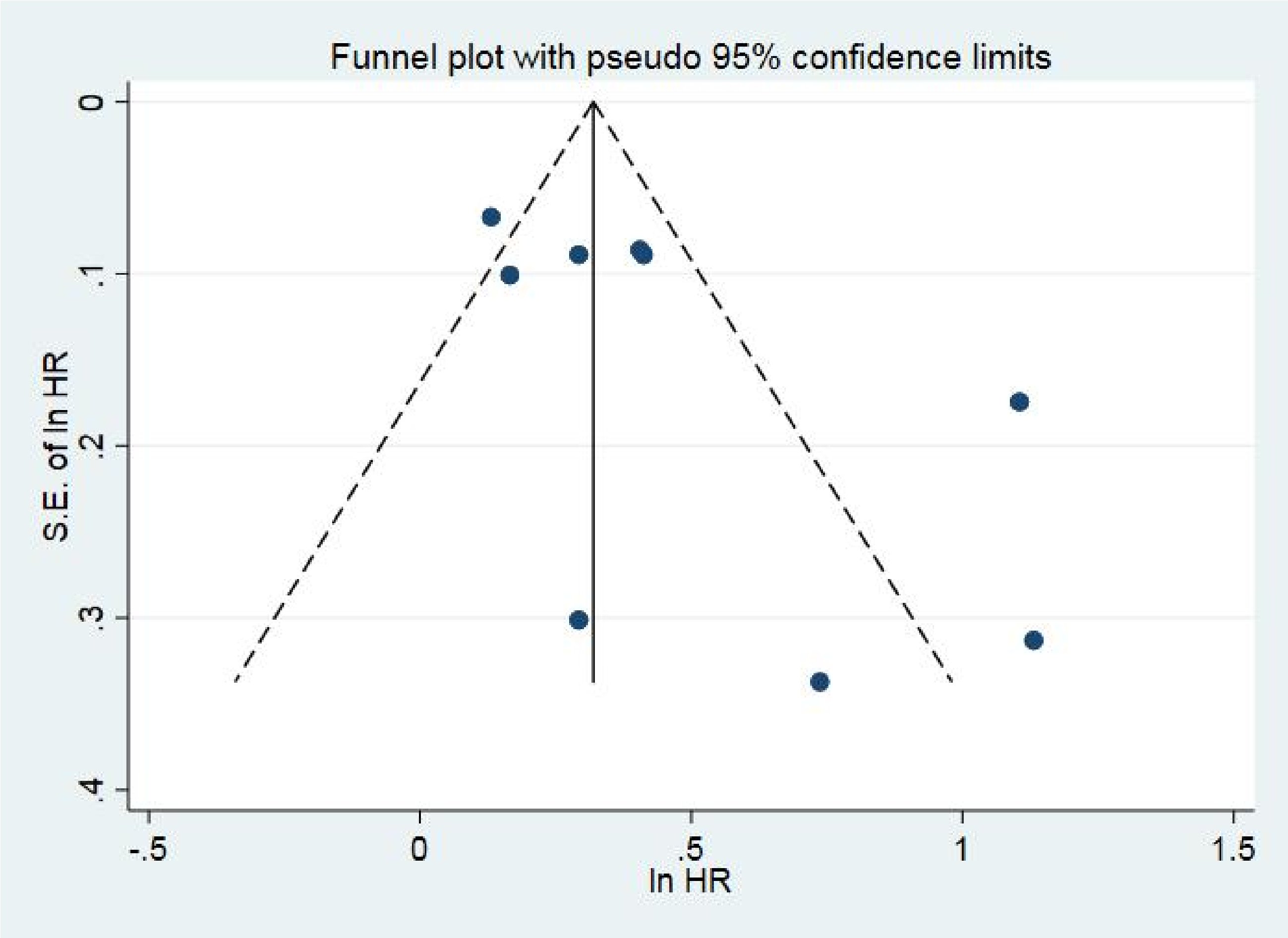

Fig. S3. Funnel plot of selected studies (NAFLD to incident hypertension) describing the relationship between effect size and standard error on the log scale. The vertical line represents the pooled effect size and the dashed lines represent the pseudo 95% confidence intervals.

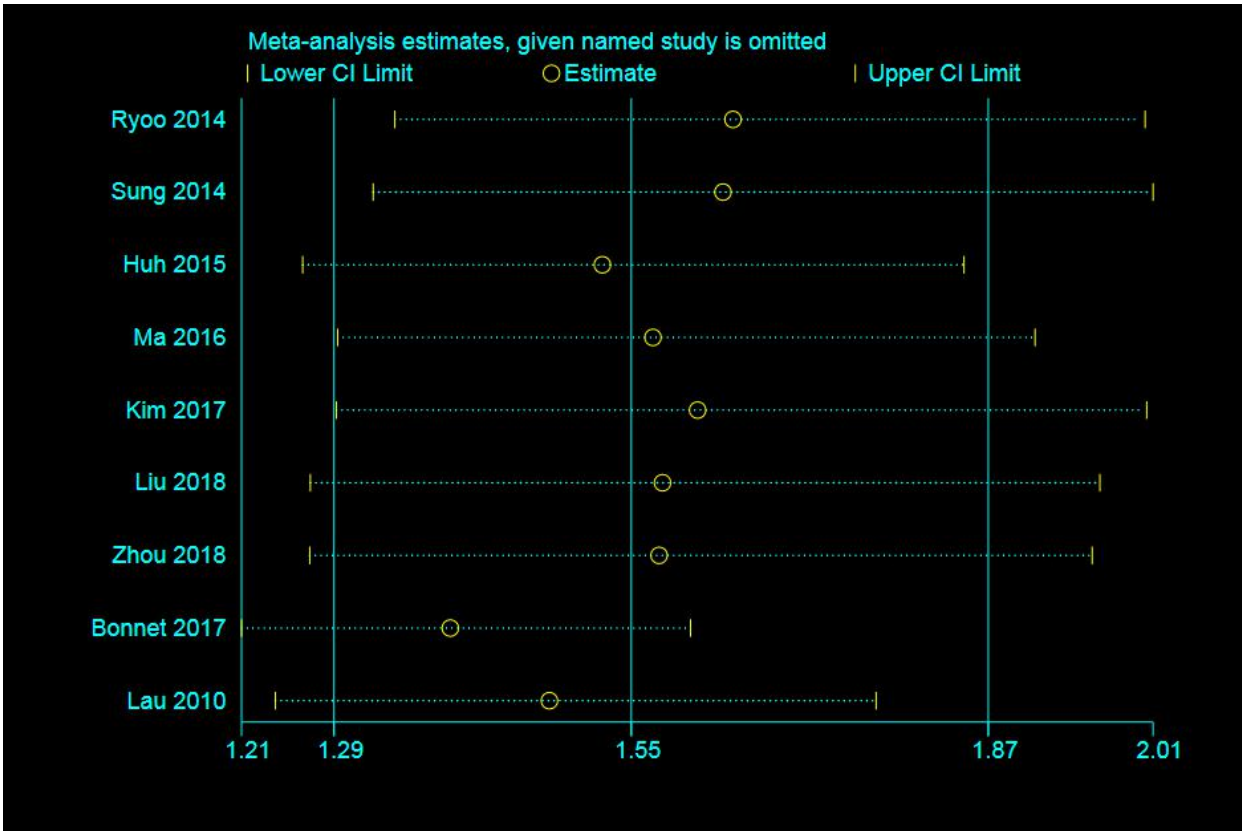

Fig. S4. Sensitivity analysis of the comparison between NAFLD and non-NAFLD on the risk of incident HTN.

Table S1. Search strategy for Pubmed and Embase

Pubmed	(("Non-alcoholic Fatty Liver Disease"[Mesh]) OR ((((((((((((Non alcoholic Fatty Liver Disease) OR (NAFLD)) OR (Nonalcoholic Fatty Liver Disease)) OR (Fatty Liver, Nonalcoholic)) OR (Fatty Livers, Nonalcoholic)) OR (Liver, Nonalcoholic Fatty)) OR (Livers, Nonalcoholic Fatty)) OR (Nonalcoholic Fatty Liver)) OR (Nonalcoholic Fatty Livers)) OR (Nonalcoholic Steatohepatitis)) OR (Nonalcoholic Steatohepatitides)) OR (Steatohepatitides, Nonalcoholic)) OR (Steatohepatitis, Nonalcoholic))) AND (("Hypertension"[Mesh]) OR ((((Blood Pressure, High) OR (Blood Pressures, High)) OR (High Blood Pressure)) OR (High Blood Pressures)))
Embase	#17. #12 AND #15 NOT 'animal experiment':ab,ti NOT 'animal':ab,ti NOT 'mouse':ab,ti NOT 'rat':ab,ti NOT 'animal model':ab,ti NOT 'meta analysis':ab,ti NOT 'systemetic review':ab,ti<br>#16. #12 AND #15<br>#15. #13 AND #14<br>#14. #9 OR #10 OR #11<br>#13. #1 OR #2 OR #3 OR #4 OR #5 OR #6 OR #7 OR #8<br>#12. 'clinical article'/exp OR 'major clinical study'/exp OR 'prospective study'/exp OR 'cohort analysis'/exp OR 'cohort':ti,ab OR 'observational':ti,ab OR 'case control':ti,ab OR 'multivariate':ti,ab<br>#11. 'high blood pressures'<br>#10. 'high blood pressure'<br>#9. 'hypertension'/exp<br>#8. 'nonalcoholic steatohepatitides'<br>#7. 'nonalcoholic steatohepatitis'<br>#6. 'nonalcoholic fatty livers'<br>#5. 'nonalcoholic fatty liver'<br>#4. 'nonalcoholic fatty liver disease'<br>#3. 'nafld'<br>#2. 'non alcoholic fatty liver disease'<br>#1. 'nonalcoholic fatty liver'/exp
